# Dominance of P-glycoprotein 12 in phenotypic resistance conversion against ivermectin in *Caenorhabditis elegans*

**DOI:** 10.1371/journal.pone.0192995

**Published:** 2018-02-23

**Authors:** Luiza Almeida Figueiredo, Thais Fuscaldi Rebouças, Sebastião Rodrigo Ferreira, Gabriela Flavia Rodrigues-Luiz, Rodrigo Cambraia Miranda, Ricardo Nascimento Araujo, Ricardo Toshio Fujiwara

**Affiliations:** 1 Department of Parasitology, Institute of Biological Sciences, Federal University of Minas Gerais, Belo Horizonte, Minas Gerais, Brazil; 2 Center of Agrarian and Biological Sciences, University of Vale do Acaraú, Sobral, Ceará, Brazil; Louisiana State University, UNITED STATES

## Abstract

While diseases caused by nematodes remains a considerable drawback for the livestock, agriculture and public health, anthelmintics drug resistance has been observed over the past years and is a major concern for parasite control. Ivermectin, initially considered as a highly potent drug, currently presents a reduced anti-helminthic efficacy, which is influenced by expression of several ATP-binding cassette transporters (ABC), among them the P-glycoproteins (Pgps). Here we present some evidences of Pgps dominance during Ivermectin resistance/susceptibility using Pgps double silencing in *C*. *elegans* and the phylogenetic relationship of Pgps among nematodes, which strengthen the use of this model for study of drug resistance in nematodes. Firstly, we evaluated the quantitative gene expression of 12 out the 15 known Pgps from resistant and WT strains of *C*. *elegans*, we demonstrated the upregulation of Pgps 12 and 13 and downregulation of all remaining Pgps in ivermectin resistant strain. By using an RNAi loss-of-function approach we observed that Pgp 12 gene silencing reverts the resistance phenotype to ivermectin, while Pgp 4 gene silencing does not alter the resistance phenotype but induces a resistance in wild type strain. Interestingly, the dual silencing of Pgp 12 and Pgp 4 expression demonstrates the dominance of phenotype promoted by Pgp 12 silencing. Finally, *in silico* analysis reveals a close relationship between Pgps from *C*. *elegans* and several nematodes parasites. Taken together, our results indicate that Pgp 12 is crucial for the resistance to ivermectin and thus a good candidate for further studies aiming to develop specific inhibitors to this transporter, allowing the continuous use of ivermectin to control the burden on animal and human health inflicted by nematode parasites globally.

## Introduction

Diseases caused by nematodes remains a considerable drawback for the livestock, agriculture and public health, contributing to increasing production costs, decreased food supply and deficits in host health [1–2]. Programs for parasite control are culturally performed through employment of anthelmintics and the improper use of these drugs for treatment of human and animal populations, like underdosing, contributes to selection of resistant parasites [3–5]. Ivermectin is an important macrocyclic lactone (ML) and the most commonly used drug to treat parasitic nematode infections of the gastrointestinal tract, the lungs, filarial infections and infestations with arthropods [6]. The ubiquitous use of this drug has led to the selection of resistance in several target species including *Cooperia oncophora* [7], *Haemonchus contortus* [8], *Ostertagia* spp. [7], *Trichostrongylus* spp. [8], *Onchocerca volvulus* [4, 9–10] and *Dirofilaria immitis* [11–12].

Previous studies have shown that ML anthelmintics can be transported by several ATP-binding cassette transporters (ABC), whose expression levels might be regulated in ML resistant nematodes [13–15]. Among these ABC transporters, the P-glycoproteins (Pgps) are known to perform cell efflux of several foreign substances [13–16] and their overexpression are related to multidrug resistance in mammalian cells and human tumor cells [17–18]. Although the relationship between Pgps and ivermectin resistance in nematodes has been shown [13, 14, 16, 19–20], here we present some evidences of Pgps dominance using Pgps double silencing in *C*. *elegans* and the phylogenetic relationship of Pgps among nematodes, which strengthen the use of this model for study of drug resistance in nematodes.

Currently it is known that *Caenorhabditis elegans* genome encodes 15 Pgps [21]. Due to the simplicity of maintenance, innocuous character and the simple life cycle that last less than 4 days [22], the *C*. *elegans* display an excellent and feasible model compared to many other nematodes, providing the opportunity to conduct different assays focusing on viability, behavior, survival, reproduction and development influenced by anthelmintic [23–25]. The aims of this study were focused to further understand the role of Pgps in ivermectin resistance by using an RNAi loss-of-function approach and determine the impact of our findings by use of *in silico* analyses to evaluate similarity among nematodes Pgps protein sequences.

## Materials an methods

### Strains and selection of ivermectin-resistant *C*. *elegans*

The wild-type Bristol N2 strain of *C*. *elegans* was kindly provided by Dr. Carlos Eduardo Winter (University of São Paulo), and cultured on nematode growth medium (NGM) with NA22 *Escherichia coli* under standard conditions[22]. Resistant worms were stepwise in vitro selection by addition of increased doses (1 ng/ml) of ivermectin (Ourofino, Brazil) to a Petri dish plate. After 1 week at 1 ng/ml ivermectin, all the survivor worms were transferred onto plates containing 2 ng/ml. Thereafter, *C*. *elegans* were transferred onto NGM plates containing higher concentrations of drug. After 48 weeks of culture, resistant *C*. *elegans* were able to grow at 30 ng/mL ivermectin (IVR30), a concentration that is lethal to the wild type.

### *C*. *elegans* L3 production

IVR30 and wild-type (WT) *C*. *elegans* were cultured over seven days in BOD incubator at 22°C, washed with M9 medium [26] and filtered through three consecutive sieves with pores of 40 μm, 30 μm, and 20 μm, respectively. L_3_ larvae retained in the 20 μm strainer were collected by backwashing and were washed by centrifugation at 700 g for 4 minutes, followed by two washes with M9 medium.

### Pgp gene expression in ivermectin-resistant strain

Worms were harvested from NGM plates and washed using M9 buffer [26]. Total RNA from worm pellets (IVR30 and WT) were extracted using RNA NucleoSpin^®^ RNA II (Macherey-Nagel, Germany) and converted into cDNA by using a Transcriptor High Fidelity cDNA Synthesis Kit (Invitrogen, USA). Quantitative PCR was performed using 50ng of cDNA, 1 μM of gene specific primers and 2X Power Sybr^®^-Green ABI (Thermo Fisher, USA) in 10μL final volume. Reactions were performed in the Applied Biosystems 7500 Real Time PCR System (Life Technologies, USA) using the following PCR cycling conditions: 95°C 10 min; 95°C 15s and 60°C 1min, for 40 cycles. Analyses were performed at 7500 Software v2.0.1 and results were determined by using 2^-ΔΔCt^ method [27]. Specific Pgps and housekeeping primers were designed using a web-based tool (Primique). Primer sequences as well as sequence confirmation PCR products are presented in Table 1.

**Table 1:** Primers and sequence information.

Genes	Primers	Sequences 5`→ 3`	Product lenght	Sequence confirmation—PCR products
Pgp 1	Foward / Reverse	TAGCACCTGAAGATGTCCTG / AATGTCGTTGTGTATCGGTAC	160pb	tagcacctgaagatgtcctgaaaaccgcaattaagactgtcgaggactatgaaggtgacaacattgattccaatggcgaaattaaaatcacaagagatgccaaagaagaggttgtaaataaggtttccattccacaattgtaccgatacacaacgacatt
Pgp 2	Foward / Reverse	AACGAAGTCAGACAAGTCTAAG / CATGAATAACTGCTGCAACTG	150pb	aacgaagtcagacaagtctaagctctcattcaagtgactcttcaatcgatgaatcaactgttaaactcacaaattatgggatattctattacactcaaggagttgatctacttcttttaattactggaacagttgcagcagttattcatg
Pgp 3	Foward / Reverse	CAGAAGACGATATAACACTTGG / AATGCTCCATTAACTGCACTC	154pb	cagaagacgatataacacttggcaaattcactccaaaaccaagtcctcaagattcttatcagggaaacttttttgatgtgtttcgagatgccgactacaaagattacatattattcagtggtggattaattttgagtgcagttaatggagcatt
Pgp 4	Foward / Reverse	ATTGCCAGCGAGCATGTGGAG / TGCCGCGCTTAGTACAAGCC	153pb	attgccagcgagcatgtggagctggggtccagacctgataagaagaagaagaaatctcgttcatcgcagggaaatagtctttccaatttgtttcgacactctggatgtgcagactatttgcttcttctaggtgggcttgtactaagcgcggca
Pgp 5	Foward / Reverse	TTAACTGGTATGTGTCAACCG / CACAACGCGATTCATATCAC	156pb	ttaactggtatgtgtcaaccgtttgaaagctatacacttggagaaacttctcaagtactggttaaagttacaaatgctattaacaataagactatagaccctgtcgatcttgcgcatgcttacaaactttttgaaagtgatatgaatcgcgttgtg
Pgp 7	Foward / Reverse	AACTGCTCAAGTCCTGGTAAC / ACATTCCGCGTTCATACTGC	101pb	aactgctcaagtcctggtaacaattacaaatgcaattaacaacaaaactattgaccctgccgatctcaaaaaagcatatgagcagtatgaacgcggaatgt
Pgp 8	Foward / Reverse	CGATGTGCTGATTCCAGTTG / ATGGTTGTGTTAATCCTGTGC	149pb	cgatgtgctgattccagttgaaaatgagaagaaaacaacgaactggacgaaattcgtgaaagtcgtgtggcaatgtacaagtaaatgggaaaagtttctgttcgtaattggagtggtttctgcaatttgcacaggattaacacaaccat
Pgp 9	Foward / Reverse	ATTCATCATCGGAAGGATCATC / CCTACTGCCAACATTAACCG	118pb	attcatcatcggaaggatcatcagagaaaaaagaagaagctccaccgccgccaaaaatctcaatttttcaactataccggtacacaagcacagtagatcggttaatgttggcagtagg
Pgp 10	Foward / Reverse	GGACTGTCGATTGCGATG / TCGAATGTATGCCTTCTTGATG	102pb	ggactgtcgattgcgatgtttattgcagctttctgccagcgaattgcttgggaaatttcgtcgatccgtcaagtgttccgcatcaagaaggcatacattcga
Pgp 11	Foward / Reverse	AGCAGTATTACGACAAGATGC / CCACGGACAAGCATACAAC	129pb	agcagtattacgacaagatgcaaactggcttgataaacattcatcagggagtatcacgtgccaactgaatgaaaacattgaagtaatttctgatggacttggaaacaaatgttgtatgcttgtccgtgg
Pgp 12	Foward / Reverse	GAAGTGGCGTAAGAATGGAG / AACCGACATCCTCAGTCG	107pb	gaagtggcgtaagaatggagatgcgatcgagccaattgatggaattccaatggagaatgggaagaagaaagatacaactgtgaatgtgtcgactgaggatgtcggtt
Pgp 13	Foward / Reverse	GTGTCTCTCAACCAGTTCTTG / ACGAAGATTCCAAGTCCTAAG	136pb	gtgtctctcaaccagttcttgcaattatctcgggtcgaatgacaaatgtgttgctagtcattgatcctttgagtaaagaatttaaaaccaaaacgatggaaaatgtatacatcttcttaggacttggaatcttcgt
CDC-42	Foward / Reverse	CTGCTGGACAGGAAGATTACG / CTCGGACATTCTCGAATGAAG	111pb	ctgctggacaggaagattacgatcgattaaggcctctatcgtatccacagaccgacgtgtttcttgtttgcttctccgtggttgctccagcttcattcgagaatgtccgag
PMP-3	Foward / Reverse	GTTCCCGTGTTCATCACTCAT / ACACCGTCGAGAAGCTGTAGA	115pb	gttcccgtgttcatcactcatctctatgacgacgtttcacctgcagaattgaatggaattgtttcacggaatgcattcttctacctgtacctcatctacagcttctcgacggtgt

### RNAi production and assay

Fragments designated for RNAi of Pgps 4 and 12 were obtained by polymerase chain reaction (PCR) from cDNA and were cloned into pGEM^®^-T Easy vector (Promega, USA). The resulting plasmids were transformed into DH5α *E*. *coli* strain. The positive DNA clones were extracted with Wizard^®^ *Plus* SV Minipreps DNA Purification System (Promega, USA) and digested with NOT I enzyme restriction (Promega, USA). The digested amplicons were cloned into L4440 feeding vector (Addgene, USA). The resulting plasmids were transformed into HT115(DE3) RNase III-deficient *E*. *coli* strain. Single colonies of HT115 bacteria containing the cloned L4440 plasmid were picked and grown in LB culture with 100 μg/mL ampicillin and 12.5 μg/mL tetracycline for approximately 2 hours at 37°C and 150 rpm until reaching OD_600_ (optical density) between 0.5–0.6. Further, 1 nM IPTG was added and bacteria was seed into NGM-agar plates previously supplemented with 100 μg/mL ampicillin, 12.5 μg/mL tetracycline and 0.4mM IPTG. Seeded plates were dried at room temperature and IVR30 and WT *C*. *elegans* L_3_ were transferred onto plates. All plates were kept in BOD incubator at 22°C for 7 days until *C*. *elegans* have consumed all bacteria. Additional control using transformed *E*. *coli* strain cultured with regular (non-supplemented with IPTG) NGM agar were used in the experiment (control-non silenced).

### Motility test

RNAi silenced (IVR30 and WT) and control-non silenced (IVR30 and WT) *C*. *elegans* L_3_ were resuspended in M9, and 1000 larvae in 100 μL of suspension were distributed on 96-well microplate (1000 larvae/well). Ivermectin was added at a final concentration of 30 ng/mL (34mM). Microplates containing ivermectin and larvae were incubated in BOD incubator at 22°C. After 48 hours, 15 μL of culture suspension containing 150 larvae was removed for further analysis and quantification of the number of paralyzed larvae using an optical microscope at 100x magnification. RNAi silenced and control-non silenced worms were cultured only in M9 medium, for negative control group (demonstrating the natural paralyses observed in a culture), and cultured in M9 medium with 1000mM of ivermectin for positive control (where worms were always 100% paralyzed). Larvae were considered paralyzed when presenting with straight body and absence of any motility [28–29].

### Propidium iodide staining

After 48 hours of incubation with ivermectin (30 ng/mL), propidium iodide (Sigma-Aldrich, USA) was added to the microplates at a final concentration of 20 μM [29–30]. Again, RNAi silenced and control-non silenced worms were cultured only in M9 medium, for negative control group, and cultured in M9 medium with 1000mM of ivermectin for positive control (The controls fluorescence emission were used to calibrate the software analysis). Microplates were incubated for 15 minutes at room temperature in a horizontal shaker at 120 rpm followed by reading at LAS ImageQuant^™^ GE 4000 with excitation in white light and emission at 605 nm. Densitometric analyses of the images were performed using the software GE Image Quant TL 8.1.

### P-glycoproteins diversity

For P-glycoprotein sequences, a search for protein nematodes available in NCBI database (National Center for Biotechnology Information) was performed using the following keywords: P-glycoprotein, PGP, multidrug resistance protein. Hypothetical and partial protein sequences were excluded. The sequences were aligned using ClustalW 2.0 software with the default parameters. To identify the clusters formed by P-glycoprotein sequences, the pairwise distance was calculated and the distance matrixes were further generated. The distances between the sequences were generated using the package PHYLIP. To provide a visual representation of each distance matrix, we used the multidimensional scaling (MDS) plot with two dimensions. The MDS and graphing were performed using the R software platform.

### Statistical analyses

The statistical analysis and graphs were made using GraphPad Prism 7.0 software (GraphPad Inc, USA) using one-way ANOVA test with Turkey’s multiple comparisons test to compare groups and One sample t test to compare the sample value to the reference number (WT basal expression). The significance level was p <0.05.

## Results

### Expression pattern of Pgps in IVR30 strain

**Fig 1:**
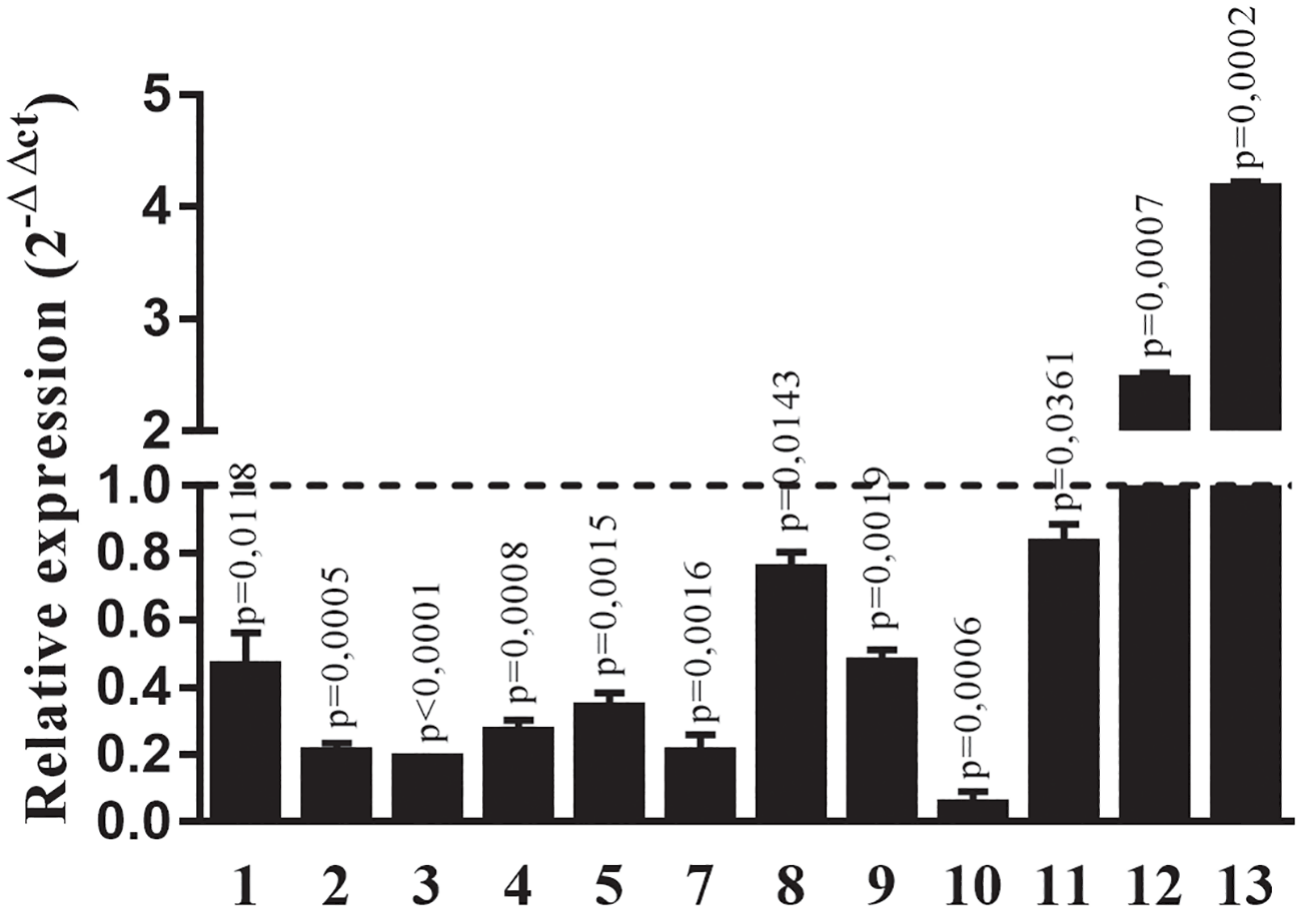
*C*. *elegans* Pgps genes profile using 2^-ΔΔCt^ method. Pgps gene expression for WT strains are represented by dashed line. P values were obtained with One sample t test, comparing sample value to WT expression reference value.

Using resistant and WT strains of *C*. *elegans* we evaluated the quantitative gene expression of 12 out the 15 known Pgps. Only the Pgps 12 and 13 were upregulated in the IVR30 in comparison with WT strain. All the other Pgps were downregulated (Fig 1).

To assess the role of Pgps during drug resistance/susceptibility to ivermectin, the expression of all Pgps were knocked down by RNAi. However, consistent results were obtained only for Pgp-4 and Pgp-12.

### RNAi-mediated Pgp 12 gene silencing reverts the resistance phenotype to ivermectin

**Fig 2:**
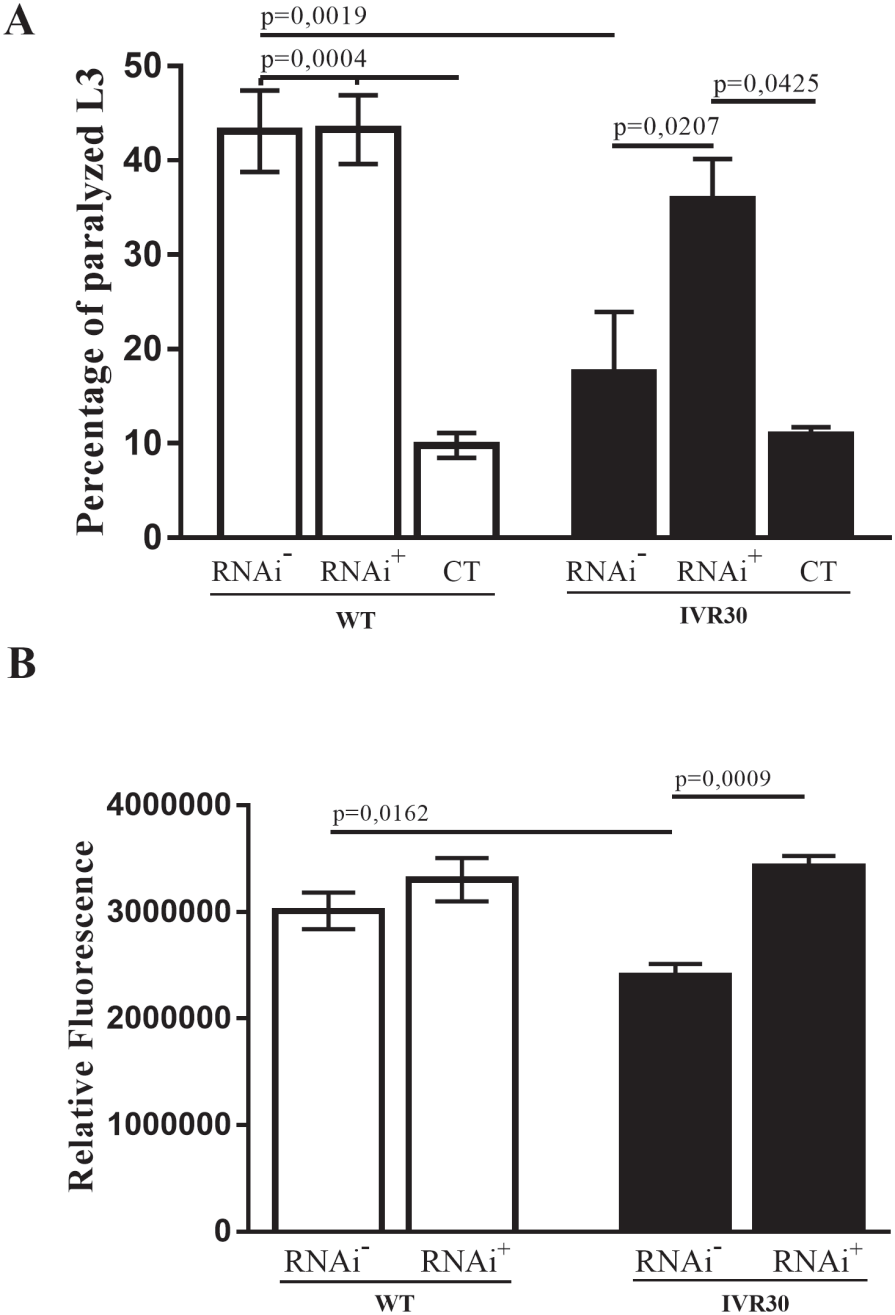
Evaluation of RNAi-mediated impairment of Pgp 12 expression in the WT and IVR30 strain. (A) Percentage of L_3_ paralysis after 48 hours of ivermectin exposure for groups Pgp 12 silenced [RNAi^+^], Pgp 12 non silenced [RNAi^-^] and negative control [CT], cultured only in M9 medium and not exposed to ivermectin treatment. (B) Assessment L_3_ viability cell using propidium iodide marker after 48 hours ivermectin exposure in Pgp 12 silenced [RNAi^+^] and Pgp 12 non silenced [RNAi^-^] groups. P value refers to one-way ANOVA test with Turkey’s multiple comparisons test.

RNAi-mediated impairment of Pgp 12 expression in the IVR30 strain induced a significant increase in worm paralysis (Fig 2A) and viability (Fig 2B), when compared to the control-non silenced worms (p = 0,0207 and p = 0,0009, respectively), after ivermectin challenge. Indeed, the paralysis and viability values of Pgp 12 RNAi-silenced worms approached those of WT strain silenced and not silenced. Gene silencing of Pgp 12 did not affect paralysis and viability of WT worms.

### RNAi-mediated Pgp 4 gene silencing does not alter the resistance phenotype but induces a resistance in wild type strain

**Fig 3:**
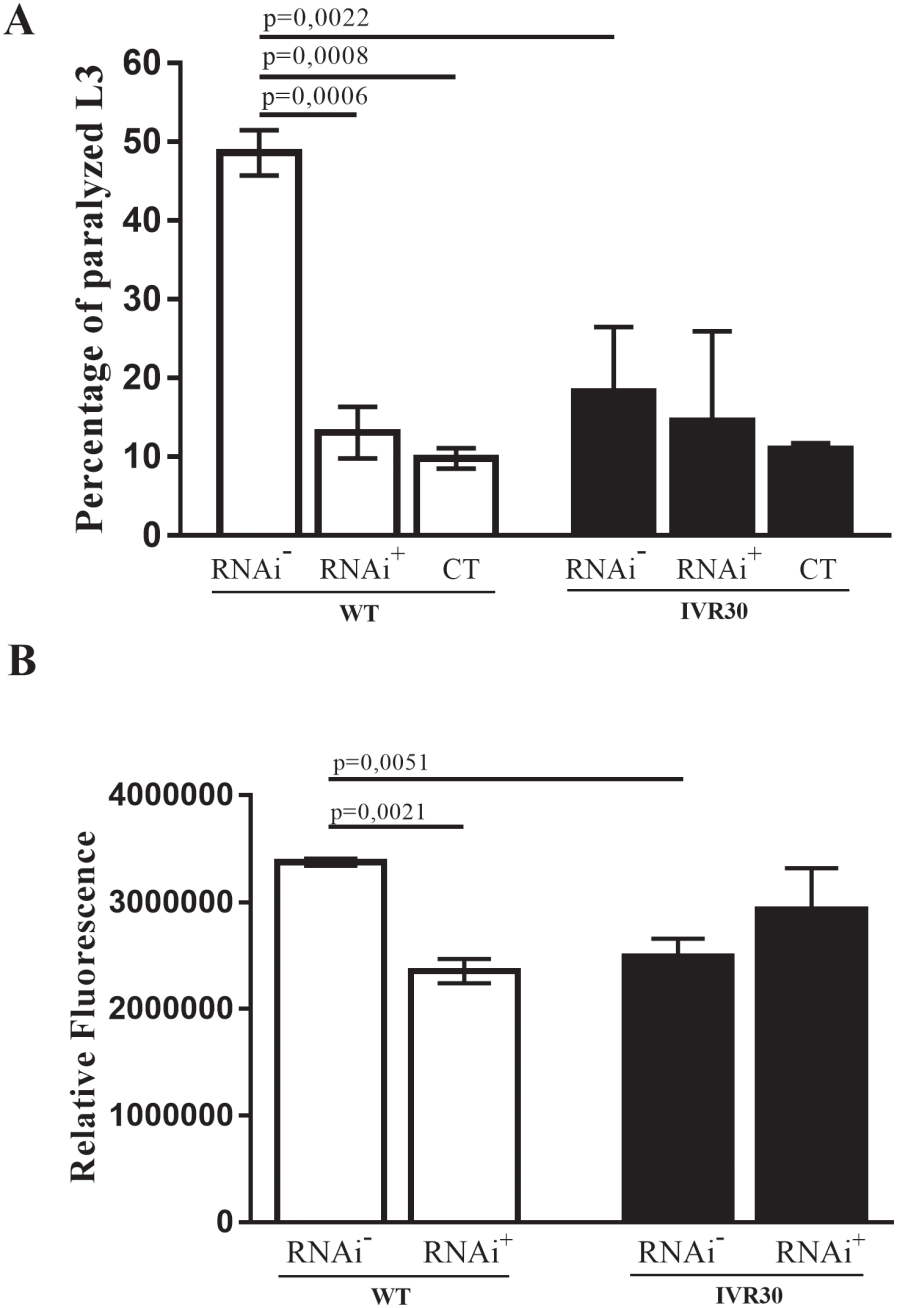
Evaluation of RNAi-mediated impairment of Pgp 4 expression in the WT and IVR30 strain. (A) Percentage of L_3_ paralysis after 48 hours of ivermectin exposure for groups Pgp 4 silenced [RNAi^+^], Pgp 4 non silenced [RNAi^-^] and negative control [CT], cultured only in M9 medium and not exposed to ivermectin treatment. (B) Assessment of L_3_ viability cell using propidium iodide marker after 48 hours ivermectin exposure in Pgp 4 silenced [RNAi^+^] and Pgp 4 non silenced [RNAi^-^] groups. P value refers to one-way ANOVA test with Turkey’s multiple comparisons test.

Following the challenge with ivermectin, no significant changes in worm paralysis (Fig 3A) and viability (Fig 3B) were observed in Pgp 4 silenced worms in comparison to the control-non silenced worms of the IVR30 strain. Interestingly, in WT strains RNAi-mediated silencing of Pgp 4 and ivermectin challenge promoted a significant decrease in the paralysis and viability of worms, therefore inducing a phenotype similar to that observed in resistant worms. The paralysis and viability between the both silenced groups (WT and IVR30) did not present any significant differences. Similarly, the results observed for Pgp 4 gene silencing in WT was equivalent to observed for the non-silenced resistant strain.

### Dual silencing of Pgp 12 and Pgp 4 expression leads to the dominance of phenotype promoted by Pgp 12 silencing

**Fig 4:**
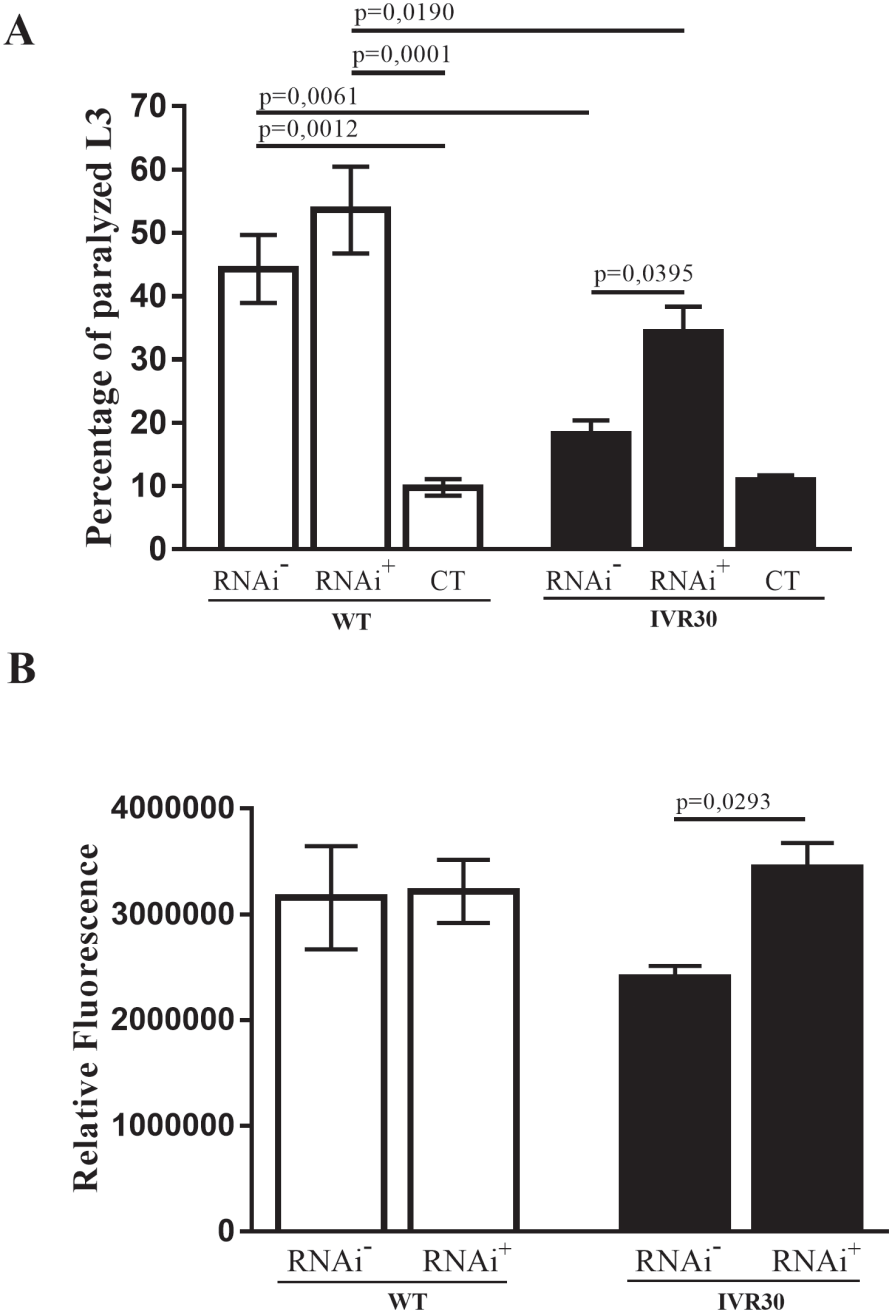
Evaluation of RNAi-mediated impairment of Pgp 4 and 12 expression in the WT and IVR30 strain. (A) Percentage of L_3_ paralysis percentage after 48 hours ivermectin exposure for groups Pgps 4 and 12 silenced [RNAi^+^], Pgps 4 and 12 non silenced [RNAi^-^] and negative control [CT], cultured only in M9 medium and not exposed to ivermectin treatment. (B) Assessement of L_3_ viability cell using propidium iodide marker after 48 hours ivermectin exposure in Pgps 4 and 12 silenced [RNAi^+^] and Pgps 4 and 12 non silenced [RNAi^-^] groups. P value refers to one-way ANOVA test with Turkey’s multiple comparisons test.

Dual silencing of Pgp 12 and Pgp 4 was performed to investigate whether these share a role in the ivermectin resistant phenotype. Following ivermectin challenge, we found a significant increase in paralysis and viability of worms in the IVR30 strain when compared to the control-non silenced group (p = 0.0395 and p = 0.0293, respectively). Significant differences in the paralysis (p = 0.0190) but not in the cellular viability was detected when the dual silenced groups (WT and IVR30) were compared. It is worth noticing that although Pgp 4 silencing affected the paralysis and viability of WT worms, dual silencing did not produce significant changes after ivermectin challenge (Fig 4).

### In silico analysis reveals a close relationship between Pgps from *C*. *elegans* and nematodes parasites

**Fig 5:**
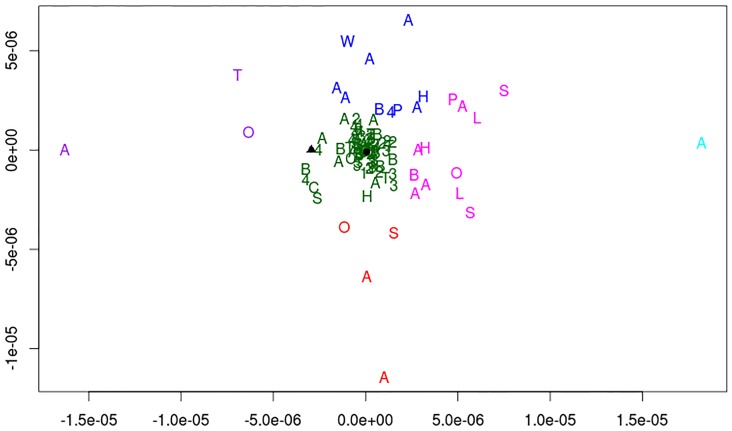
*In silico* analysis revealing a close relationship between Pgps from *C*. *elegans* and nematodes parasites represented. As follow: *Ascaris suum* (A), *Brugia malayi* (B), *Cooperia oncophora* (C), *Haemonchus contortus* (H), *Loa loa* (L), *Onchocerca volvulus* (O), *Parascaris equorum* (P), *Strongyloides ratti* (S), *Trichuris trichiura* (T), *Wuchereria bancrofti* (W), *C*. *brenneri* (1), *C*. *briggsae* (2), *C*. *remanei* (4), Pgp 4 of *C*. *elegans* (Δ), Pgp 12 of *C*. *elegans* (•), all remaining Pgps from *C*. *elegans* (2).

A phylogenetic approach was performed using protein sequences of Pgps from different organisms including helminthes parasites with medical relevance to investigate the level of similarity among them (Fig 5). Protein sequence cluster analysis indicate that *C*. *elegans* Pgps are highly similar to the Pgps of nematodes parasites such as *Ascaris suum* (A), *Brugia malayi* (B), *Cooperia oncophora* (C), *Haemonchus contortus* (H), *Loa loa* (L), *Onchocerca volvulus* (O), *Parascaris equorum* (P), *Strongyloides ratti* (S), *Trichuris trichiura* (T) and *Wuchereria bancrofti* (W).

## Discussion

Initially considered as a highly potent drug, ivermectin has now a reduced anti-helminthic efficacy and therefore research on the possible mechanisms of resistance are important, especially in the light of the negative effects of diseases caused by parasitic nematodes in animal [31–32] and in human [33–34] healthcare. Previous studies revealed the involvement of Pgps in the development of ivermectin resistance in various nematodes [16, 19–20], including the free-living nematode, *C*. *elegans* [35–37]. We have confirmed some of these results and extended the study on the role of Pgps in ivermectin resistance developed by *C*. *elegans* to show that Pgp 4 might be directly related to susceptible phenotype and that Pgp 12 directly related to resistant phenotype in this worm. In addition, we show that Pgps 1 to 13 of *C*. *elegans* have a close phylogenetic relationship to Pgps of other parasitic nematodes.

In our results we observed that expression of most Pgps were downregulated when compared with WT whereas Pgps 12 and 13 were upregulated in an ivermectin-resistant *C*. *elegans* strain (IVR30). In contrast, Yan et al. [38] found that most of the 15 Pgps they analyzed were upregulated in their ivermectin-resistant strain (IVR10). We hypothesized that the divergence between the results may be due to difference of resistant strains selected, as in the study cited above they obtained a 10 ng/mL ivermectin-resistant strain and we selected a 30 ng/mL ivermectin-resistant *C*. *elegans* strain. In both WT and IVR30 we silenced Pgp 4 and Pgp 12 that were characteristically downregulated and upregulated, respectively. The RNAi method has been widely used in *C*. *elegans* to study gene function [39]. Interestingly, the results for paralysis and cellular viability observed for the Pgp 12 gene silencing in IVR30 strain were similar to those observed in both silenced or non-silenced WT strains. Therefore, it indicates that RNAi-mediated Pgp 12 gene silencing in IVR30 reverted the resistant phenotype to the WT, suggesting that this Pgp may be directly related to ivermectin resistance phenotype. Two studies using several *C*. *elegans* Pgps knockout strains observed that deletion of Pgp 12 resulted in increased sensitivity to ivermectin [35–36], corroborating our results.

The Pgp 4 gene silencing in IVR30 strain did not lead to a phenotypic changes after ivermectin exposure. However, such gene silencing promoted a significant reduction of paralysis and increased cellular viability in the WT strain, which rendered a phenotype observed in the resistant IVR30 strain. Furthermore, the RNAi-mediated Pgp 4 gene silencing in IVR30 did not alter the resistance phenotype but induced resistance in the WT strain, suggesting that Pgp 4 may be directly related to ivermectin susceptibility. In contrast, previous results with *C*. *elegans* Pgp 4 knockout strains showed a slight increase in sensitivity to the drug in the WT. However, other authors [38] using a high concentration of ivermectin (20 ng/mL) found that the RNAi-mediated silencing of Pgp 4 led to downregulation of this gene and a concomitant increase in the motility of the WT strain of *C*. *elegans*, a finding that is similar to the effect reported in this study.

To date no study have describe the double-silencing of nematode Pgps. In our results, the dual silencing of Pgp 12 and Pgp 4 expression in the IVR30 strain lead to increase of worm paralysis and reduction of cellular viability after exposure to ivermectin. However, the results obtained for the dual Pgp 12 and Pgp 4 silencing were similar to those obtained when Pgp 12 was silenced alone, suggesting that dual silencing of Pgp 12 and Pgp 4 expression in the IVR30 strain led to the dominance of the phenotype promoted by Pgp 12 silencing. Moreover, dual silencing of these genes did not alter the susceptibility status of the WT strain. It is possible that silencing of both Pgps in the WT strain generated a compensation phenotype effect so that the resistant phenotype, generated by Pgp 4 silencing was suppressed by Pgp 12 silencing phenotype.

To assess whether the roles ascribed for the Pgps in *C*. *elegans* resistance to ivermectin could be extrapolated to parasite nematodes, one first step is to evaluate similarity of these Pgps within the phylum Nematoda. The spatial projection allows a global view of the closest sequences (closest in spatial projection points) and the most distant (farthest points in the projection), allowing the analysis of the distribution (diversity) and the formation of groups. The distances between the closest and farthest points can be used to infer phylogenetic trees, diversity and divergence between sequences [40]. The protein sequence cluster analysis performed indicates that all *C*. *elegans* Pgps studied herein are highly similar to the Pgps of various nematodes parasites (Fig 5).

Our study is the first to perform dual silencing and reveal a compensation mechanism of one Pgp over another to modulate *C*. *elegans* resistance status. We are also the first to analyze phylogenetically the Pgps and show that they are highly similar in many of the nematodes of medical and economical relevance.

## Conclusion

In conclusion, here we show that Pgp 12 is a good candidate for further studies aiming to develop specific inhibitors to this transporter so that ivermectin can still be used to control the burden on animal and human health inflicted by nematode parasites globally.
